# Flow–solid coupling analysis of underground reinforced concrete forked pipe enclosing rock and structure

**DOI:** 10.1038/s41598-023-44148-w

**Published:** 2023-10-08

**Authors:** Ming Xiao, Qingteng Yuan, Binxin Zhao, Liang Deng

**Affiliations:** State Key Laboratory of Water Resources Engineering and Management, Wuhan University, Wuhan, 430072 Hubei China

**Keywords:** Civil engineering, Energy infrastructure

## Abstract

The stability of the surrounding rock for the construction excavation and the reinforcement of the lining structure during operation in the water-rich area is a difficult problem for the design of high-head underground turnout pipes. Firstly, according to the mechanism of excavation load release and surrounding rock damage evolution, the seepage effect of excavation in the construction of the forked caves is coupled to the surrounding rock stress damage, and an iterative method of numerical simulation of the coupled mutual feedback effect of excavation surrounding rock stress and seepage is proposed. Then, based on the cracking characteristics of the high internal water pressure reinforced concrete turnpike lining, a numerical analysis method of the coupling interaction between lining cracking and internal water seepage is proposed by coupling internal water seepage to stress damage in the lining by cracking the forked pipe structure. Applying the aforementioned method to a forked pipe project, the results show that: during the construction period, there is a significant increase in the damage zone, stress, and displacement of the rock around the cavern after considering the coupled iterations; during the operation period, with the increase in internal water pressure, the lining structure accelerates cracking due to the external infiltration of internal water; after the internal water is applied, the surrounding rock bears the main internal water pressure and the reinforcement bears only part of the circumferential force. The method provides theoretical support for the analysis and calculation of the reinforcement of similar underground high-pressure tunnels for rock support and lining structures and has certain theoretical and engineering significance.

## Introduction

With the increasing construction of large hydropower stations and pumped storage power stations, there is a rising demand for high-head, large-diameter underground forkpipe structures. However, the geological environment in which the fork cavern envelope is placed is becoming more complex, making it challenging to design the pipe lining structure according to current specifications and theories. A reasonable analysis of the structural forces of reinforced concrete turnouts under complex conditions of rock stability and high internal water pressure is an important part of ensuring the safe and reliable operation of underground reinforced concrete turnout structures, efficient and environmentally friendly construction, and high-quality and economic engineering.

The design of an underground reinforced concrete forkpipe structure involves the interaction of rock, concrete, groundwater, reinforcement, and other structural materials, due to the complex spatial structure and the fugitive environment, affected by the coupling of stress and seepage, its force characteristics have obvious inhomogeneity, non-linearity and irreversibility^1^, only the correct analysis of construction excavation surrounding rock stress deformation and water-filled operation lining stress cracking leading to seepage and stress coupling of reinforced concrete forkpipe structure bearing capacity characteristics, in order to provide a reasonable basis for the design of underground reinforced concrete forkpipe structure. In 1986 Schleiss^2^ proposed the permeable lining theory for hydraulic tunnels, arguing that the design of hydraulic tunnels should be based on the surrounding rock and that there is a conditional joint bearing effect between the lining and the surrounding rock. Based on the permeable lining theory, many scholars have derived some analytical expressions that consider the seepage effect and the cracking behavior of the lining. Fernandez^3^ derived an equation for calculating the effective stress in the rock surrounding of a hydraulic tunnel, taking into account the effect of radial seepage; Zongli^4^ derived an analytical formula for the radius of the plastic zone in deeply buried tunnels considering seepage and analyzed the effect of the head difference between the inside and outside of the tunnel on the stress distribution and plastic zone of the tunnel by means of an example; Changguang^5^ derived analytical formulas for stress distribution and displacement in tunnels based on the unified strength theory and softening model, considering the combined effects of rock strain softening, shear expansion, and seepage; Based on an elastic-brittle damage model, Kang^6^ derived an expression for the stress distribution and plastic zone radius of the surrounding rock under the action of internal water and verified the effect of seepage loading on the expansion of the plastic zone radius. Based on the principle of effective stress in porous media, according to the relationship between permeability and porosity and volumetric strain, Wu et al.^7,8^ established a damage evolution model and differential elastoplastic principal structure equations regarding volumetric strain and realized the whole process of hydraulically fully coupled surrounding rock stress damage based on ABAQUS; Li et al.^9^ proposed a coupled seepage-stress-damage algorithm for permeable liners based on the concrete plastic damage model and permeable lining theory; Zhongqiu et al.^10^ carried out a fully coupled analysis of the peritectic rock and lining of the deeply buried diversion tunnel of Jinping II Hydropower Station based on the theory of equivalent saturated porous media, treating rock and concrete as elastic–plastic damage materials and considering the respective deformation characteristics and dynamic evolution of permeability coefficients; Yafeng et al.^11^ used the equivalent permeability coefficient based on the cubic theorem and the pipe sheet joint tension to reflect the effect of structural deformation on the permeability coefficient, thus proposing an equivalent coupling model for pipe sheet lining; Based on the continuous medium large deformation theory and damage mechanics theory, Shanpo et al.^12^ introduced the plastic damage evolution and seepage mutual coupling method to the Mohr–Coulomb criterion and established a coupled elastic–plastic large deformation seepage-stress model for clay rocks; Zhou et al.^13^ developed a coupled water-force model in FLAC3D to monitor displacements and pore pressures in different directions around the tunnel throughout the construction period and to determine the depth of impact of excavation on deformation and pore pressure; Ma et al.^14^ proposed a fully coupled flow-deformation model for saturated fractured porous media under elastic–plastic damage; Yi et al.^15^ developed a new fully coupled fluid flow and rock damage model for simulating the extension of hydraulic fractures in porous media; Simon et al. used the convergence-constraint method to convert the three-dimensional coupled problem into a two-dimensional plane strain model to study the effect of transient H–M coupling on deep saturated rock masses. These theoretical studies have effectively illustrated some of the mechanisms of coupled stress and seepage in hydraulic tunnels, but the underground high-pressure reinforced concrete turnouts are subject to complex environments, staggered spatial mechanical behavior, and irreversible cyclic seepage and stress coupling, and their stress mechanisms and structural safety assessment constitute a difficult design challenge for underground reinforced concrete turnouts.

In order to comprehensively understand the working conditions of the construction and operation of underground reinforced concrete turnouts and to ensure their construction quality and operational safety, this paper uses theoretical derivation and numerical simulation to establish an analysis method for underground high-pressure concrete turnouts, considering the coupling effect of seepage and stress. By studying the mutual feedbacks between excavation damage and external water infiltration in turnouts and between concrete lining cracking and internal water infiltration, the method of evaluating the safety of excavation stability of underground turnouts and the idea of calculating the reinforcement of turnout structure lining according to the analysis of permeable lining are proposed, which provides better theoretical significance and engineering value for the design of similar underground reinforced concrete turnouts.

## Coupled analysis method of stress and seepage in perimeter rock for underground cavern construction and excavation

When the groundwater is richer or the reservoir storage level is raised, resulting in increased groundwater activity, the stability of the surrounding rock is not only affected by the excavation release load but also by the role of external water infiltration. With the cavern chamber surrounding rock in the excavation release stress and the coupling of external water infiltration, the permeability of the surrounding rock accelerated, damage radicalization and seriously affecting the quality of the excavation of the underground chamber. Therefore, a reasonable evaluation of the stability of the surrounding rock under the coupling action of seepage and stress in underground cavern chambers is the key to giving full play to the bearing capacity of the surrounding rock and ensuring the safety of the underground cavern lining structure. Exploring the mechanism of coupled action of seepage effect and stress release outside the construction excavation underground cavern and reasonable analysis methods is a key issue for the design and construction of complex underground space bifurcated tunnel structures.

### Method of analysis for construction excavation of underground cavern enclosures

Microfractures will occur when the principal tensile strain $\varepsilon_{1}$ of the rock exceeds the ultimate tensile strain $\left[ \varepsilon \right]$ of the rock when the excavation releases the load. The ultimate tensile strain of a rock mass is currently difficult to determine experimentally and can generally be determined from engineering experience by using the ratio of the compressive strength $R_{a}$ of the rock to the modulus of elasticity $E$^16^:

1 $$[\varepsilon ] = R_{a} /(K \cdot E)$$

where $K$ is the coefficient of influence on the characteristics of the surrounding rock, which can be determined from the ratio of compressive and tensile strength of the surrounding rock. When the rock microfractures appear, the stress will be damaged, resulting in a reduction in the strength parameters of the material and a non-linear change in the stress–strain relationship. According to Frantziskt and Desai^17^, when $\varepsilon_{1} > \left[ \varepsilon \right]$, the damage stress in the rock mass can be determined by introducing a damage factor $D$ according to the following equation:

2 $$\sigma_{ij}^{D} = \left( {1 - D} \right)\sigma_{ij} + \frac{D}{3}\sigma_{ij} \delta_{ij}$$

where $\delta_{ij}$ is the Kronecker Delta function; $\sigma_{ij}$ is the stress value consistent with the elastic–plastic constitutional relationship; and $D$ is the damage internal variable describing the extension of microfractures in the rock, which can be calculated from the plastic strain deflection tensor after the rock enters plastic failure according to the following equation^18^:

3 $$D = D_{n} \left[ {1 - \exp \left( { - K\xi^{a} } \right)} \right]$$

where $D_{n}$, $K$, $a$ are the damage material constants, determined from material tests, and $\xi = \sqrt {e_{ij} \cdot e_{ij} }$ $e_{ij}$ is the plastic deviatoric tensor.

During the excavation load release process, the plastic load of the excavation is calculated in a graded iteration^19^, and at each level of the plastic load iteration, the rock damage coefficient value $D$ calculated according to Eq. (3) can be substituted into Eq. (2), and the plastic damage stress matrix can be derived by deducing that

4 $$\left[ {H_{D} } \right] = \left( {1 - D + D\delta_{ij} /3} \right)\left[ {H_{p} } \right] + \left( {D - D\delta_{ij} /3} \right)\left[ {H_{e} } \right]$$

where $\left[ {H_{p} } \right]$ and $\left[ {H_{e} } \right]$ denote the plastic and elastic stress matrices of the rock mass, respectively. Before each level of plastic load increment is applied, the damage stiffness matrix $\left[ {K_{D} } \right]$ is modified according to (1) to discriminate whether or not the element enters damage according to Eq. (1), and the damage stiffness matrix $\left[ {K_{D} } \right]$ is simply modified for elements that enter damage by the following equation for iterative calculations:

5 $$\left[ {K_{D} } \right] = \int {\left[ B \right]^{T} \left[ {H_{D} } \right]\left[ B \right]dv}$$

During each level of plastic load increment $\left\{ {\Delta R_{{p_{i} }} } \right\}$ iteration, the plastic damage stiffness can be kept constant by iterating the displacement increment resulting from the level i of plastic load increment according to the following equation:

6 $$\left[ {K_{e} } \right]\left\{ {\Delta \delta_{i} } \right\}_{j} = \left\{ {\Delta R_{{p_{i} }} } \right\} + \left[ {K_{D} } \right]\left\{ {\Delta \delta_{i} } \right\}_{j - 1} + \left\{ {\Delta P^{s} } \right\}^{e}$$

where $\left\{ {\Delta P^{s} } \right\}^{e}$ denotes the seepage load generated by the change in element infiltration head, which can be calculated according to Eq. (13) based on the change in seepage generated by each level of plastic load increment.

### Iterative analysis method for rock seepage in construction excavations

Groundwater loading is one of the main loads in hydraulic tunnels, and when the permeability boundary of groundwater changes after excavation of the chamber, external water infiltration will occur in the cavern. The change in groundwater actually only moves in the wet zone below the free water surface, however the free water surface is generally unknown in practical engineering problems and needs to be solved using iterative solutions. In this paper, the seepage problem on the wet region $\Omega_{w}$ is transformed into a new boundary value problem in the whole domain $\Omega$ by redefining the form of variational inequality^20^:

7 $$v = - k\nabla \varphi + v_{0}$$

where $v$ is the seepage velocity, v_0_ is the initial flow velocity, k is the second-order permeability tensor, $\nabla$ is the gradient operator, $\varphi = z + p/\gamma_{w}$ is the total head, z is the vertical coordinate component,$p$ is the pore water pressure, and $\gamma_{w}$ is the bulk density of water. The initial flow velocity $v_{0}$ is introduced to eliminate the spurious seepage field on the dry zone $\Omega_{d}$ and is expressed as:

8 $$v_{0} = H\left( {\varphi - z} \right)k\nabla \varphi \quad H\left( {\upphi - z} \right) = \left\{ {\begin{array}{*{20}l} 0 \hfill & {if\;\upphi \ge z\;\left( {in\;\Omega_{{\text{w}}} } \right)} \hfill \\ 1 \hfill & {if\;{\upphi < }z\;\left( {in\;\Omega_{{\text{d}}} } \right)} \hfill \\ \end{array} } \right.$$

In the finite element method analysis, a theoretically rigorous Signorini type variational inequality method is used to solve for the seepage based on the principle that the seepage satisfies the continuity equation and seepage boundary conditions, and the SVA method (a combination of substructure, variational inequality, and adaptive penalty Heaviside function) is established for iterative calculations. The method theoretically eliminates the singularity of the exfiltration point and the resulting mesh dependence, and is able to precisely locate the exfiltration point and free surface of a strongly non-linear seepage problem. After the finite element discretization, the iterative format of the SVA method of seepage analysis can be expressed as a finite-dimensional trial vector space $\Phi_{V}^{h}$ in which a vector $\phi^{j + 1} \in \Phi_{V}^{h}$ such that for $\forall \psi \in \Phi_{V}^{h}$, all of which have:

9 $$\left( {\psi - \phi^{j + 1} } \right)^{T} K\phi^{j + 1} \ge \left( {\psi - \phi^{j + 1} } \right)^{T} q^{j + 1}$$

where $K = \sum\nolimits_{e} {\iiint_{{\Omega_{e} }} {B^{T} KBd\Omega }}$, $q^{j} = \sum\nolimits_{e} {\iiint_{{\Omega_{e} }} {B^{T} v_{0}^{j} d\Omega }}$, $j$ is the free surface iteration step,$e$ is denoted as the finite element grid cell summation, and $B$ is the geometric matrix of the finite element model. According to the basic principle of finite element discretization, the three-dimensional finite element seepage iterative equation of Eq. (9) can be simplified in the $j$ step of the seepage iteration calculation as:

10 $$\left[ A \right]\left\{ H \right\} = \left\{ F \right\}$$

where $\left\{ H \right\}$ the nodal head column vector, $\left\{ F \right\}$ the nodal infiltration load obtained by seepage boundary integration, and $\left[ A \right]$ the element seepage conduction matrix calculated from the rock permeability coefficient $k$. The following strict convergence control criteria were used for each step of the seepage calculations:

11 $$\sum {\Delta H_{i} } < \varepsilon_{1} \sum {H_{i} } \;and\;\Delta H_{\max } < \varepsilon_{2} H_{\max }$$

where $\Delta H_{i}$ is the water head difference between node $i$ in two adjacent free surface iterations,$H_{i}$ is the water head of node $i$ in the current iteration step;$\Delta H_{\max }$ is the maximum water head difference for each node in two adjacent free surface iterations,$H_{\max }$ is the upstream water level, $\varepsilon_{1}$ and $\varepsilon_{2}$ are specified tolerances, which are generally taken as $\varepsilon_{1} = 10^{ - 6}$ and $\varepsilon_{2} = 10^{ - 5}$^20^.

### Coupled seepage and stress calculation method for construction excavation rock

During the calculation of the excavation of the underground forked pipe chamber, iterative calculations of the coupled surrounding rock stress and external water infiltration are carried out in the following steps:

1. Firstly, based on the plastic loads released by the excavation, iterative calculations of the deformation of the surrounding rock excavation are carried out for each level of plastic release loads according to Eq. (6); then the stresses and damage factors in the surrounding rock resulting from the deformation of the perimeter rock for each level of plastic release loads are calculated according to Eqs. (2) and (3);
2. Calculation considering the effect of damage evolution on the permeability coefficient of the surrounding rock based on the stress and damage coefficient of the surrounding rock. According to the empirical equation of permeability coefficient versus positive stress from Louis^21^ drilling pressure experiments, the permeability coefficient of the rock mass changes abruptly after the surrounding rock enters plastic and suffers cracking damage, so the permeability abruptness coefficient $\xi_{i}$ is introduced to describe the effect of damage evolution on seepage in the surrounding rock:
  12 $$K = \xi_{i} k_{0} e^{{b\sigma_{i} }}$$
  where $k_{0}$ is the initial permeability coefficient of the surrounding rock; $b$ is the coupling coefficient determined by experiment;$\sigma_{i}$ is the effective stress of element; the permeability jump factor $\xi_{i}$ is taken according to the stress state of the surrounding rock: 1 in the elastic stage, 5 in the plastic stage, and 100 in entering damage cracking.
3. Recalculate the permeability matrix $\left[ A \right]$ for the surrounding rock based on the permeability coefficient calculated in Eq. (12) after the excavation iteration, and calculate the change in the seepage field by iterating in Eq. (10).
4. Based on the plastic release load at each level, the difference in permeate head $\Delta H_{i}$ at each node before and after loading is calculated by iteration of Eq. (10) and the change in stress at each element $\left\{ {\Delta \sigma } \right\}$ before and after loading is calculated by iteration of Eq. (6), and then the change in permeate load and stress load at each element at the nodes resulting from the change in seepage and stress in the surrounding rock are calculated separately by the following equation:
  13 $$\text{Seepage}\;\text{variation}\;\text{loads}:\;\left\{ {\Delta P^{s} } \right\}^{e} = \iiint_{\Omega } {\left[ N \right]^{T} \left\{ {\Delta f^{s} } \right\}dxdydz}$$
  14 $$\text{Stress}\;\text{change}\;\text{loads}:\;\left\{ {\Delta P^{\sigma } } \right\}^{e} = \iiint {\left[ B \right]^{T} }\left\{ {\Delta \sigma } \right\}dxdydz$$
  where $$\left\{ {\Delta f^{s} } \right\} = \left[ {\begin{array}{*{20}c} {\Delta f_{x} } & {\Delta f_{y} } & {\Delta f_{z} } \\ \end{array} } \right]^{T}$$ is the infiltration load component in the three coordinate directions calculated by the difference in infiltration head $\Delta H_{i}$ at each node within the element before and after the iteration, $\left[ N \right]$ is the element shape function, and $\left[ B \right]$ is the element geometry matrix.
5. Superimpose the seepage change load calculated in Eq. (13) and the stress increment load calculated in Eq. (14) onto the next level of plastic load $\left\{ {\Delta R_{{P_{i + 1} }} } \right\}$, and carry out the iterative calculation of the stress and seepage coupling for the next level of plastic load according to Eq. (6) and the above iterative calculation steps.

The iterative calculation according to the above method can not only clearly describe the process of damage evolution of the surrounding rock damage by construction excavation load but also reasonably respond to the influence of seepage on the development of damage evolution of the surrounding rock and the characteristics of internal seepage of water outside the cavern excavation due to stress changes after the coupling and mutual feedbacks of seepage changes and surrounding rock stress. The method is characterized by good iterative convergence stability and fast coupling calculation speed.

## Coupling analysis method of underground fork pipe lining structure force and internal water infiltration

The design of underground high-pressure forks has undergone a conceptual shift from crack-resistant design to crack-limiting design and finally to the current permeable lining design as the internal water pressure borne by hydraulic tunnels has increased^22^. The permeable lining design means that under high internal water pressure, the concrete lining cracks to produce internal water infiltration, and the permeable lining only bears the water pressure of the head difference inside and outside the lining, with the vast majority of the internal water pressure being borne by the surrounding rock^23^. Therefore, the stress state of the underground fork lining structure and the seepage characteristics of the internal water infiltration after lining cracking are the keys to the design of the reinforcement of the underground high-pressure fork lining structure.

### Force analysis method for underground fork pipe lining structures

Due to the poor tensile properties of the concrete structure, it is easy to crack the concrete lining along the annulus under the internal pressure of the underground high-pressure fork pipe lining during operation. It is assumed that the cracked lining only transmits radial load, the annulus load is mainly borne by the reinforcement, and the lining only transmits radial load to the surrounding rock, so the thin-layer element structure is used between the surrounding rock and the concrete lining to react to the joint effect of the lining and the surrounding rock. The stiffness matrix of the thin-layer element along the local coordinates perpendicular to the lining plane (normal) and parallel to the lining plane (tangential) is:

15 $$\left[ {K^{\prime } } \right] = \left[ {\begin{array}{*{20}c} {K_{n} } & {} & {} \\ {} & {K_{st} } & {} \\ {} & {} & {K_{st} } \\ \end{array} } \right]$$

where $K_{n}$ can be taken according to the surrounding rock resistance of the forked pipe chamber structure; $K_{st}$ is taken as 0.005–0.01*K*_*n*_ for calculation stability depending on the concrete lining cracking.

The reinforcement within the concrete lining structure is modeled using a reinforcement element implicitly embedded in the concrete. The reinforcement is mainly responsible for the annular load transfer after the concrete lining is cracked. The element stiffness $\left[ {K_{s} } \right]$ of the reinforcement along the lining annulus can be superimposed on the concrete element according to the finite element equivalence principle, and the additional stiffness $\left[ {K_{f} } \right]$ generated in the concrete element can be calculated according to the following equation^24^:

16 $$\left[ {K_{f} } \right] = \left[ N \right]^{T} \left[ {K_{s} } \right]\left[ N \right]$$

where $\left[ N \right]$ is the stiffness equivalence matrix between the reinforcement element and the concrete element. When the concrete is not cracked, the reinforcement and concrete do not produce slippage, the two deformations are coordinated, and the stiffness along the radial direction of the reinforcement can be calculated as per the rod element $\left[ {K_{s} } \right] = AE_{s} /L$ ($A$, $E_{s}$ and $L$ represent the area, elastic mode, and action length of the reinforcement, respectively).When the concrete produces cracking damage, the reinforcement and concrete produce irreversible deformation sliding. In this paper, the Mazars damage model is used to describe the damage behavior of concrete materials. According to the Lemaitre equivalent force assumption, the damage coefficient of concrete materials after cracking is:

17 $$D_{c} = \left\{ {\begin{array}{*{20}l} 0 \hfill & {\varepsilon_{{eq \le \varepsilon_{D} }} } \hfill \\ {1 - \frac{{\varepsilon_{D} \left( {1 - A_{T} } \right)}}{{\varepsilon_{eq} }} - \frac{{A_{T} }}{{\exp \left[ {B_{T} \left( {\varepsilon_{eq} - \varepsilon_{D} } \right)} \right]}}} \hfill & {\varepsilon_{{eq > \varepsilon_{D} }} } \hfill \\ \end{array} } \right.$$

where $\varepsilon_{eq} = \sqrt {\varepsilon_{1}^{2} + \varepsilon_{2}^{2} + \varepsilon_{3}^{2} }$ is the equivalent strain, $0.5 \times 10^{ - 4} < \varepsilon_{D} < 1.5 \times 10^{ - 4}$ is the concrete damage strain threshold, $A_{T}$ and $B_{T}$ are the test curve fitting constants for normal concrete, $0.7 < A_{T} < 1.0$ and $10^{4} < B_{T} < 10^{5}$^18^.

The possible crack width of the lining elements can be estimated from the non-linear iterative calculation of the concrete element strain value $\varepsilon_{c}$ according to the following equation (13):

18 $$W_{m} = 4t_{e} (1 - \rho + n\rho )D_{c} \varepsilon_{c} /(\eta - \eta \rho + n\rho D_{c} )$$

where $t_{e} = 0.725 \sqrt[3]{{A_{s} d}}$, $A_{{\text{s}}} = 2dr$, $d$ is the protective thickness of the reinforcement,$r$ is the reinforcement spacing, $n = E_{s} /E_{c}$ is the ratio of reinforcement to the concrete spring mode. $\rho$ is the reinforcement ratio, $\eta = 1 - D_{c}$.

### Coupled analysis method of cracking stress and seepage in underground fork pipe lining

After the excavation of the underground turnpipe chamber is completed, the turnpipe lining and reinforcement structure are imposed on it, and the overall structural permeability matrix $\left[ A \right]$ is firstly reorganized according to the permeability coefficient of the surrounding rock after excavation and the initial permeability characteristics of the concrete structure, and the structural permeability load $\left\{ F \right\}$ is calculated by taking the head acting at the inner boundary of the turnpipe lining as the initial head boundary, and the initial permeability field during the operation of the turnpipe is calculated according to Eq. (10); Then the local coordinate stiffness of the thin element between the surrounding rock and the concrete lining calculated according to Eq. (15);The local coordinate stiffness of the implied reinforcement element calculated according to (16).The element stiffness in local coordinates $\left[ {K_{e} } \right]$ is converted to the element stiffness in overall coordinates $\left[ {K_{E} } \right]$ according to the local coordinate to overall coordinate conversion relationship matrix $\left[ R \right]$:

19 $$\left[ {K_{E} } \right] = \left[ R \right]^{T} \left[ {K_{e} } \right]\left[ R \right]$$

The overall structural elastic stiffness $\left[ {K_{e} } \right]$ is then reorganized according to the structural properties of the surrounding rock, concrete, reinforcement and thin-layered elements. Iterative calculations of the coupled seepage and stress of the lining structure are carried out according to the following equation:

20 $$\left[ {K_{e} } \right]\left\{ {\Delta \delta } \right\}_{j} = \left[ {K_{D} } \right]\left\{ {\Delta \delta } \right\}_{j - 1} + \left\{ {\Delta F_{w} } \right\}_{j - 1} + \left\{ {\Delta F_{s} } \right\}_{j - 1} + \left\{ {\Delta F_{g} } \right\}_{j - 1}$$

where $\left[ {K_{D} } \right]$ the damage stiffness of the surrounding rock into the plastic damage element, calculated according to (6); $\left\{ {\Delta F_{w} } \right\}$ is the internal water pressure increment acting on the inner surface of the fork pipe lining; $\left\{ {\Delta F_{s} } \right\}$ is the infiltration load caused by the change in the seepage field;$\left\{ {\Delta F_{g} } \right\}$ is the additional load caused by the force deformation of the reinforcement,$j$ denotes the number of iterations. The specific calculations are carried out according to the following iterative steps:

1. The first step $j = 1$,the lining structure has just been applied, no rock entry damage taken $\left\{ {\Delta \delta } \right\}_{0} = 0$; reinforcement has not yet functioned taken $\left\{ {\Delta F_{g} } \right\}_{0} = 0$; the initial seepage field node seepage head has not changed taken $\left\{ {\Delta F_{s} } \right\}_{0} = 0$; The total head $H$ acting on the inner surface of the lining is weighted into $m$ levels applied to the inner surface of the lining, and the surface load at each level is calculated according to the following formula:
  21 $$\left\{ {\Delta F_{w} } \right\}_{j} = \left( {1 + m - j} \right)\gamma H/\left( {1 + 2 + \cdots + m} \right)$$
2. The surface load of the level $j$ of Eq. (21) is substituted into Eq. (20) to iteratively calculate the displacement of each node, and the strain and stress of each element are calculated according to the basic theory of finite elements, and then the surrounding rock damage coefficient $D$ and concrete lining damage coefficient $D_{c}$ are calculated according to Eqs. (3) and (17) respectively. The damage stiffness $\left[ {K_{D} } \right]$ of the damaged element is calculated according to Eq. (6) based on the damage coefficient $D$ of the rock element. Based on the concrete damage coefficient $D_{c}$, the load increment $\Delta F_{g}$ resulting from the deformation of each reinforcement can be calculated by using the principle that the reinforcement and the concrete bear the load in proportion to the concrete damage:
  22 $$F_{g} = D_{c} E_{s} A_{s} \left( {1 - \rho + n\rho } \right)\varepsilon_{c} /\left( {\eta - \eta \rho + n\rho D_{c} } \right)$$
  where $E_{s}$ and $A_{s}$ are the rebar elastic modulus and rebar area; n is the ratio of rebar to concrete elastic modulus; $\varepsilon_{c}$ is the concrete strain value at the rebar element;$\rho$ is the reinforcement ratio, $\eta = 1 - D_{c}$. The additional nodal load $\left\{ {\Delta F_{g} } \right\}_{j}$ for the deformation of the reinforcement can be found by superimposing all the reinforcement deformation load increments and equating them to the nodes of the element in which they are located.
3. For concrete linings entering damage cracking, the crack width $W_{m}$ of the concrete structure is estimated according to Eq. (18). The permeability of concrete increases dramatically with the creation of cracks. Based on the concrete damage and permeability relationship test proposed by Picandet^23^, the permeability coefficient of the cracked element along the crack direction is calculated from the cubic law of hydraulics based on the crack opening width as:
  23 $$k_{c} = \frac{1}{{1 + 8.8\left( {\frac{\Delta }{{2W_{m} }}} \right)^{1.5} }} \times \frac{{\gamma W_{m}^{2} }}{12\mu }$$
  where $\gamma$ and $\mu$ are water gravity and dynamic viscosity coefficients respectively; $W_{m}$ is the average crack width; $\Delta$ is the crack roughness. The principal permeability coefficient calculated in (23) is then converted into a three-dimensional permeability coefficient matrix in integral coordinates according to the transformation of the cracking direction of the concrete element to the principal coordinate axis $\left[ R \right]$ by the following equation:
  24 $$\left[ {K_{Z} } \right] = \left[ R \right]^{T} \left[ {K_{c} } \right]\left[ R \right]$$
4. Firstly, the change in permeability coefficient after cracking of concrete calculated in Eq. (24) is recombined with the permeability matrix of the whole structure $\left[ A \right]$, and then the change in seepage field is recalculated according to Eq. (10); the difference between the infiltration head at each node and the infiltration head calculated in the previous iteration $\left\{ {\Delta H} \right\}$ is then calculated based on the recalculated seepage field; the change in infiltration load due to concrete cracking $\left\{ {\Delta F_{s} } \right\}_{j}$ is then calculated based on the change in head difference between the before and after seepage iterations.
5. Substitute the rock damage stiffness $\left[ {K_{D} } \right]$ calculated in the iteration of j, the additional load $\left\{ {\Delta F_{g} } \right\}_{j}$ generated by the steel deformation, the infiltration load $\left\{ {\Delta F_{s} } \right\}_{j}$ caused by concrete cracking, and the incremental surface load $\left\{ {\Delta F_{w} } \right\}_{j}$ of the fork pipe lining into the Eq. (20), and recalculate the fork pipe lining force; then repeat steps (2)–(5), when the whole load is applied and calculated until the damage element of the rock no longer increases, the additional load generated by the steel deformation, and the infiltration load caused by concrete cracking change to a certain standard, and the whole iteration of internal water infiltration calculation is completed.

The iterative calculations according to the above method not only provide a clear description of the cracking process of the fork pipe lining but also provide a reasonable response to the force characteristics of the permeable lining after being subjected to internal and external water pressure, providing a better analysis method for the structural force calculation of the high-pressure fork pipe.

## Example analysis of fork cavern surrounding rock stability and fork pipe lining structure force

**Figure 1:**
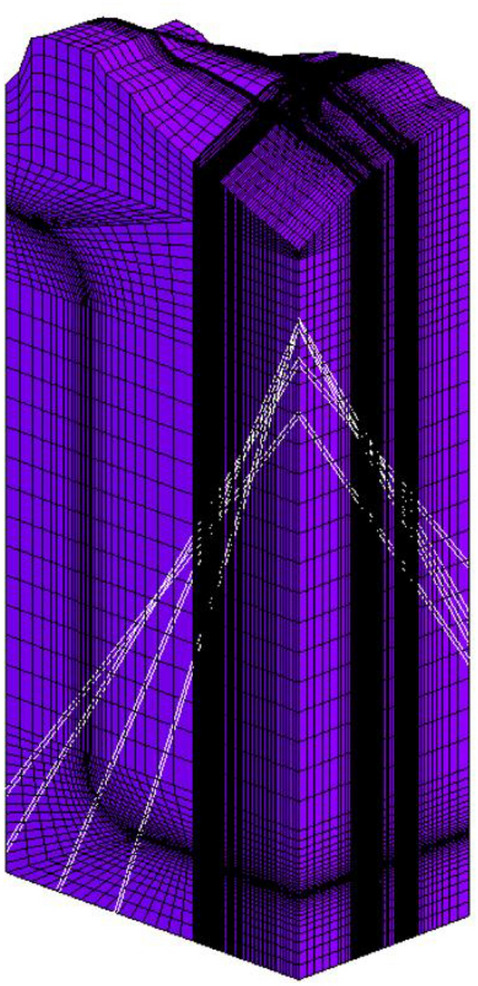
3D structural model of the forked pipe.

**Figure 2:**
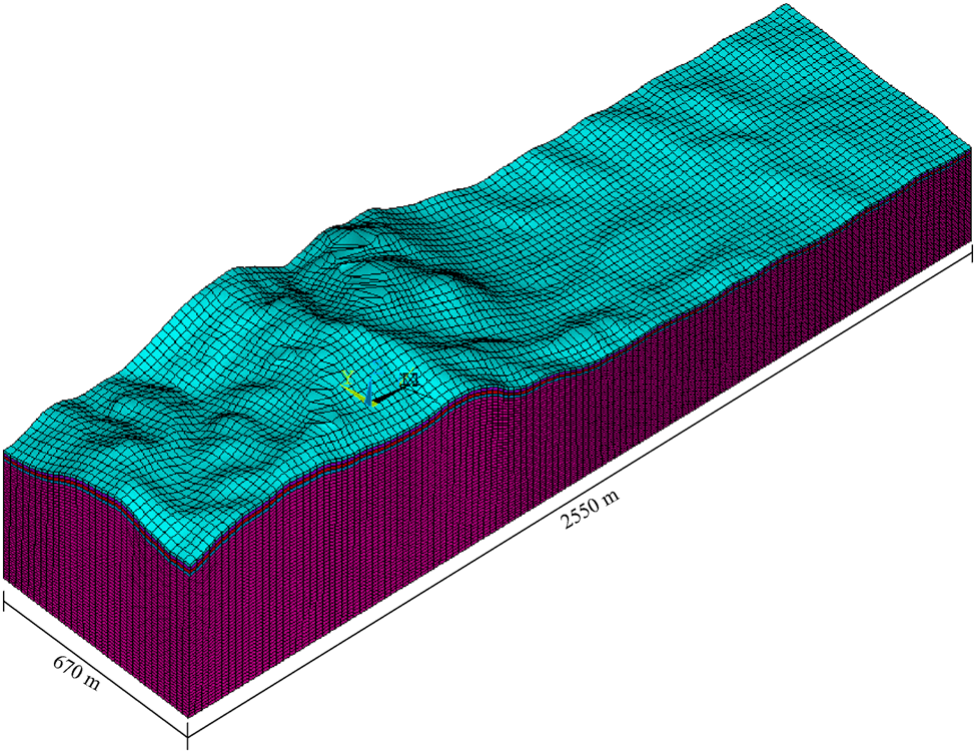
Finite element model of the 3D initial seepage field inversion calculation of the forked pipe.

In a pumped storage power station, the excavation of the turnpipe tunnel was carried out in a head-type arrangement, and the three-dimensional finite element model established according to the structural arrangement of the high turnpipe was divided into 498,034 isoparametric elements with 8 nodes (see Fig. 1), including 15,960 lining elements and 9,120 excavation elements, and the lining was made of C25 reinforced concrete with a thickness of 1.0 m. The scope and coordinate system of the finite element model are: the origin of the coordinates is located at the center of the cross section of the high pressure fork pipe section; the X-axis points downstream along the axis of the hole, the range is from − 124.81 m to 58.78 m, 183.59 m long; the Y-axis is perpendicular to the direction of water flow from the 1# unit section to the 4# unit section; the range is from − 68.70 to 46.00 m, 114.70 m long; the Z-axis coincides with the geodetic coordinates; the range is from − 29.07 to 380.00 m. The initial ground stress field was calculated based on the measured values of three ground stress points. The initial ground stress field was calculated based on the inversion of the measured ground stress values at three points, and the first principal stress value at the high pressure fork pipe was distributed between − 9.79 and − 11.27 MPa, the second principal stress was distributed between − 8.43 and − 9.21 MPa, and the third principal stress was distributed between − 6.83 and − 7.78 MPa; The initial seepage boundary of the structural calculation model was calculated based on the established 3D finite element. The initial seepage boundary of the structural calculation model was calculated based on the established three-dimensional finite element inversion model, including the upstream and downstream reservoir ranges (see Fig. 2). An inversion calculation of the initial seepage field over a large area of the project area based on a given upstream and downstream constant head boundary and some long-view bore water level values; The surrounding rock at the fork pipe is class III, with modulus of deformation *E* = 8.6 GPa, Poisson’s ratio $\mu = 0.25$, friction coefficient $f = 1.2$, cohesion *C* = 1.45 MPa, rock permeability coefficient $k = 5 \times 10^{ - 7} \;\text{m}/\text{s}$; The concrete has a volume weight of 25.0 kN/m^−3^, a modulus of elasticity of 28GPa, a Poisson's ratio of 0.167, a compressive strength of 11.9 MPa and a tensile strength of 1.27 MPa. The anchored support parameters: $\Phi 25@1.5 \times 1.5\;\text{m}$ plume-shaped staggered arrangement, *L* = 4.5 m, spray layer $\delta = 15\;\text{cm}$.

The high-pressure fork pipe is buried at a large depth, and there is high groundwater action during the construction period. The external head of water in the fork cavern area is about 334 m, and the design value of internal water pressure in the fork pipe during the operation period is 483 m. The coupling of internal water infiltration and stress during the construction and operation periods is investigated.

### Coupling of seepage effects and stresses outside the excavation of the fork in the construction excavation analytical calculations

**Figure 3:**
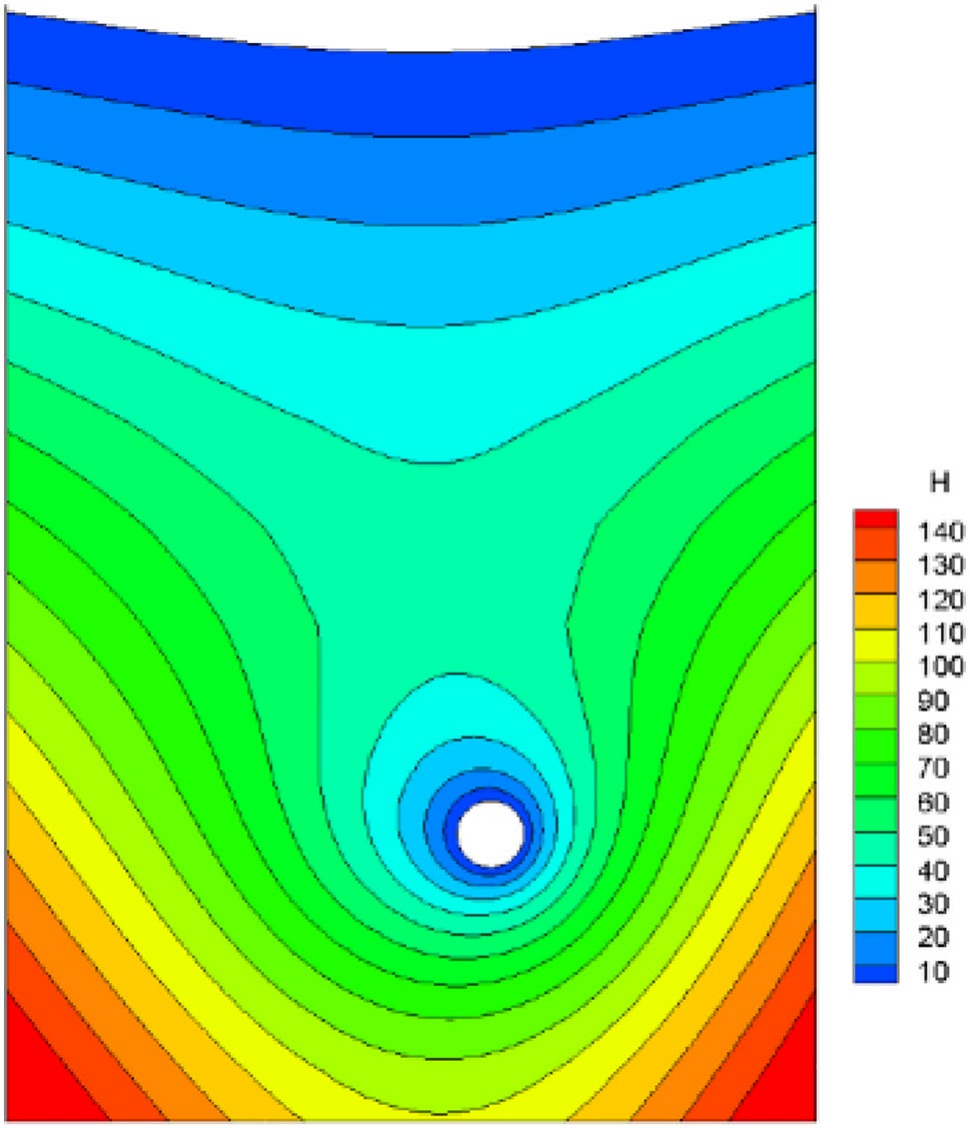
Seepage field of the excavated forkhole.

**Figure 4:**
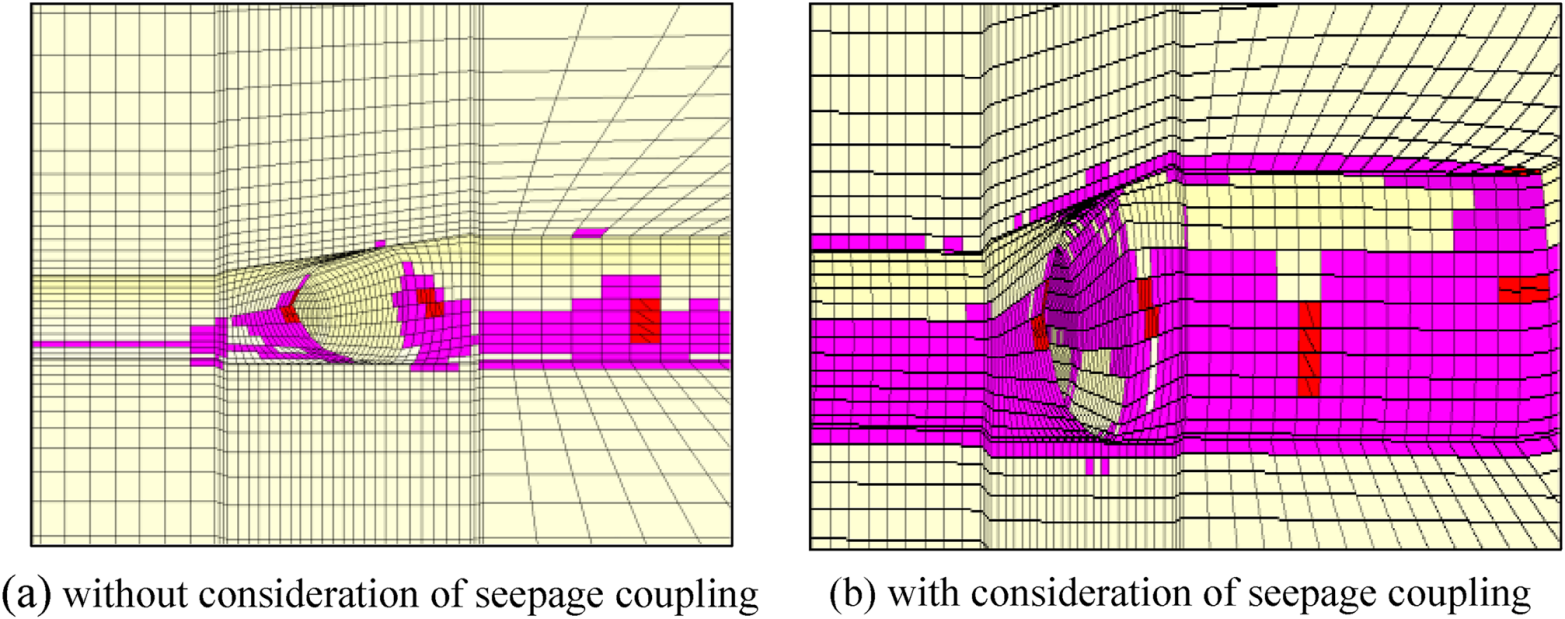
Forkhole damage zone.

**Figure 5:**
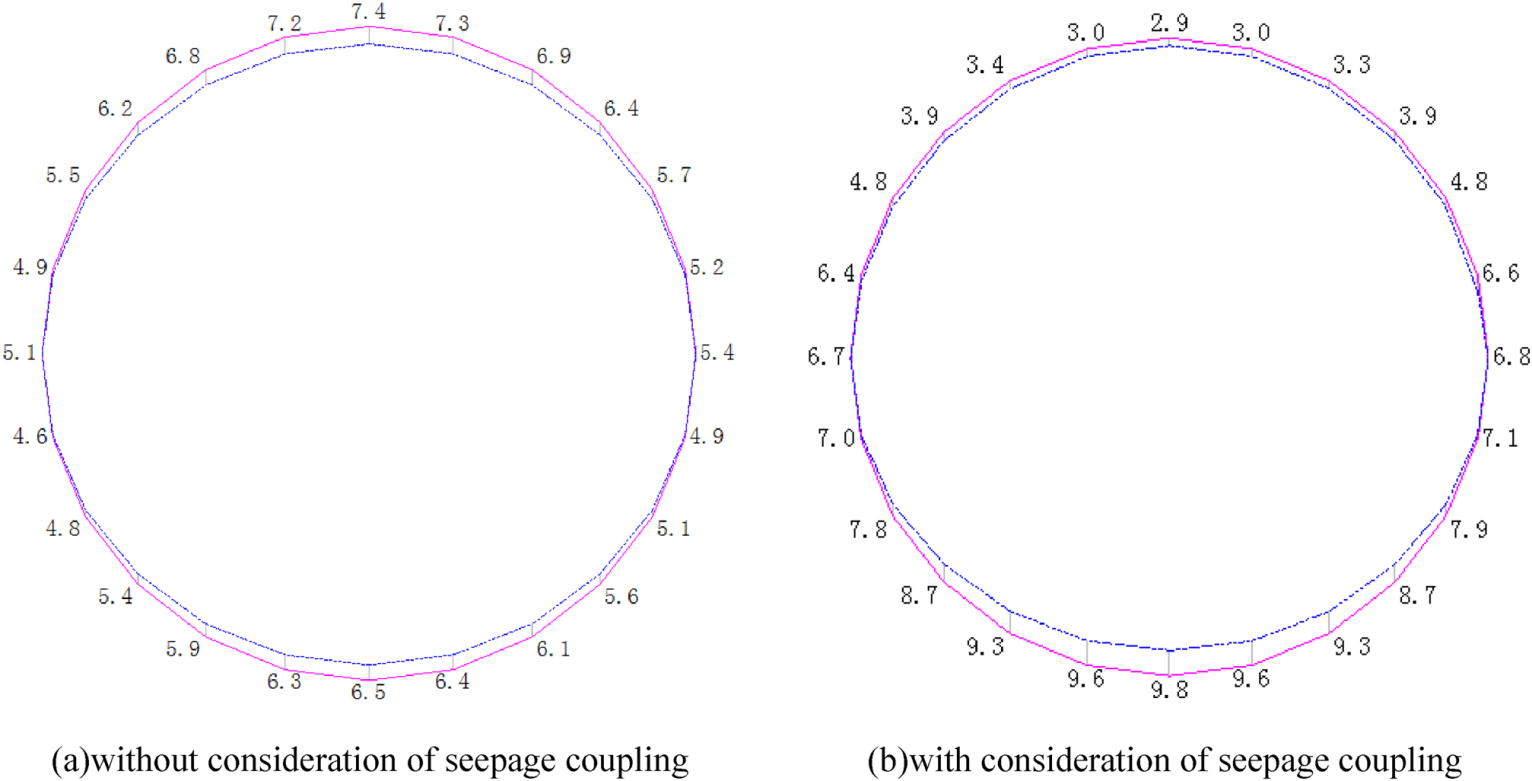
Displacement of forkhole.

**Figure 6:**
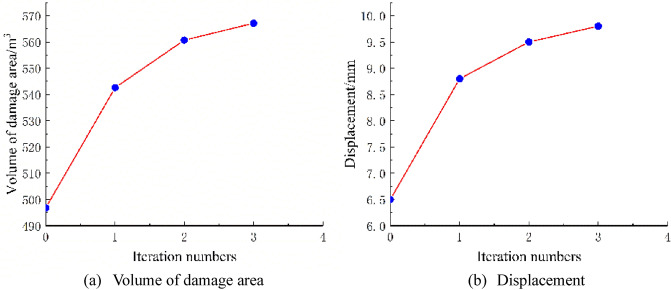
Trend with number of iterations.

**Figure 7:**
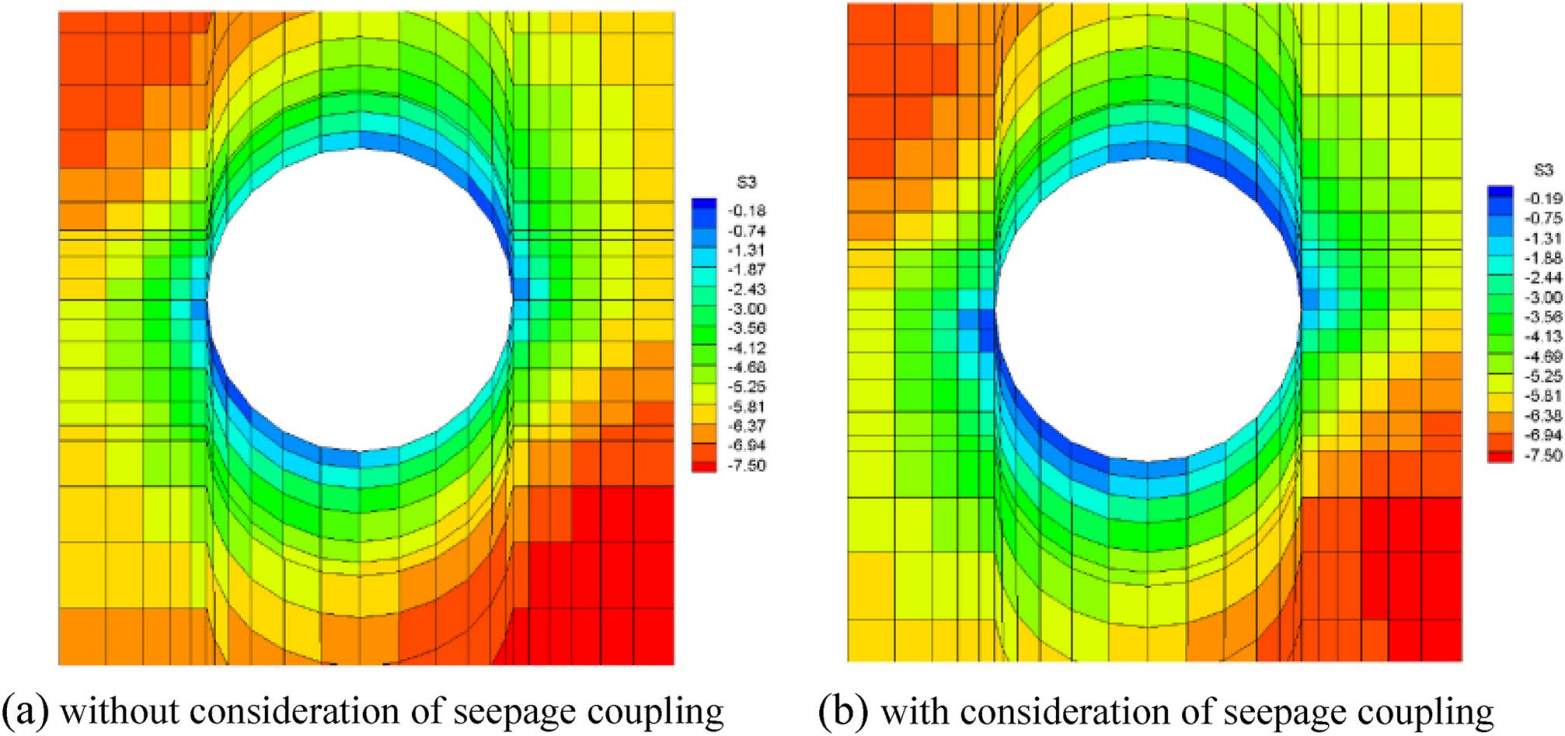
Third principal stress of forkhole.

**Figure 8:**
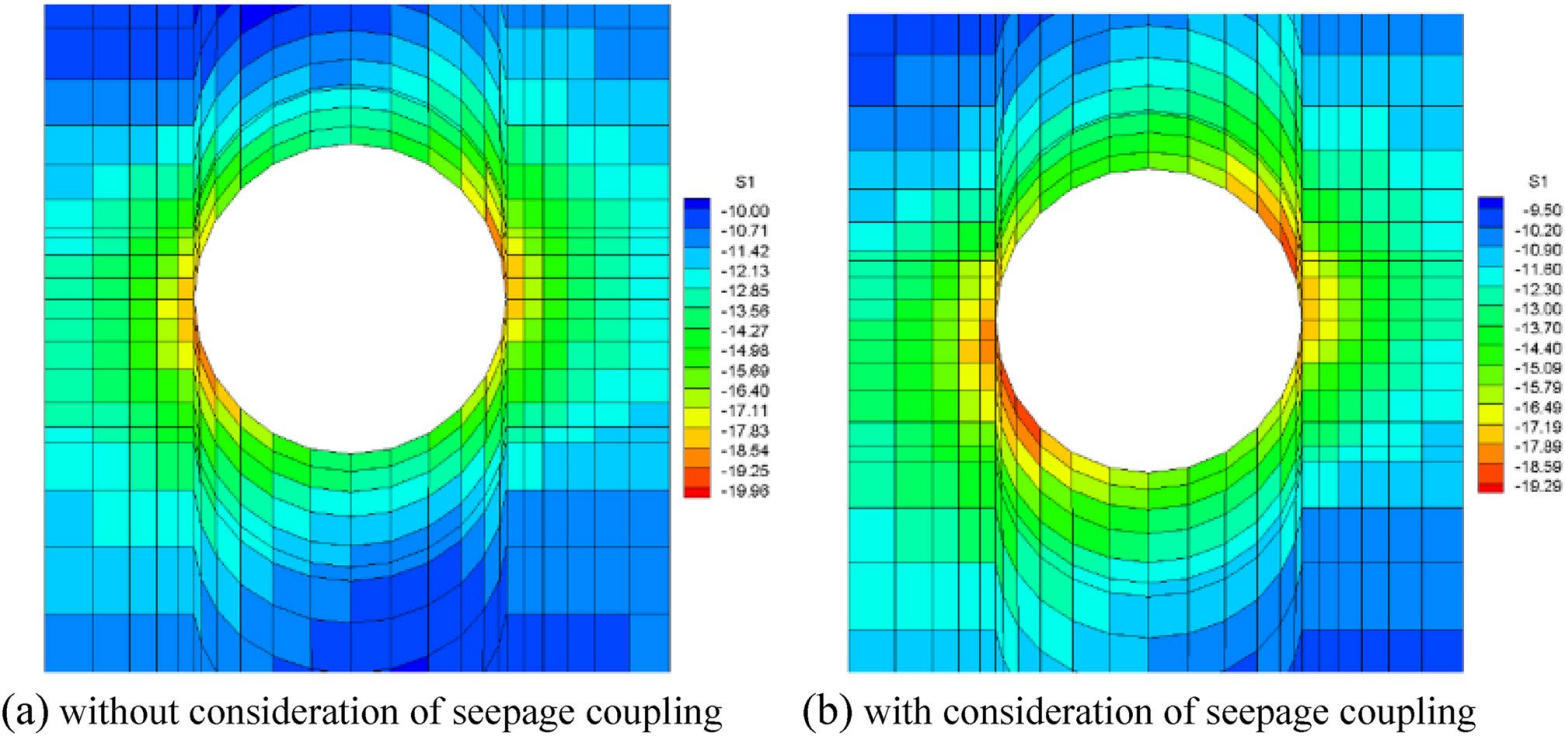
First principal stress of forkhole.

It is assumed that the fork pipe excavation will be completed in a single pass due to the small extent and volume of the fork pipe excavation. In order to illustrate the consideration of seepage and stress coupling effects, a comparative analysis of the results of two types of excavation with and without consideration of seepage and stress coupling was carried out, respectively, from which the following patterns can be seen:

1. Based on the results of the initial inversion calculation of the seepage field over a large area of the project area (Fig. 2), the water level at the downstream right boundary of the structural calculation model (Fig. 1) was obtained as 191.58 m and the water level at the upstream left boundary of the model as 277.86 m. The inversion seepage head values were applied to the structural calculation model boundary, and the excavation boundary of the turnpipe tunnel was regarded as the overflow boundary of the seepage. In the seepage field of the construction excavation of the turnpipe tunnel, iterative calculations were carried out. From the iterative calculation process, it appears that the forked tunnel excavation seepage flow and stress, coupled with a reduction in the original groundwater level around the forked tunnel, also increase the rock damage around the hole. After the excavation of the fork in the tunnel was completed, the pressure head at the cavern face was reduced to zero, and the closer to the tunnel, the lower the pressure head became. The groundwater level in the vertical direction of the tunnel center line was reduced from 100 m in the initial state to 85 m (Fig. 3). This indicates that the excavation of the fork in the tunnel, the change in stress in the surrounding rock, and the seepage flow coupling are detrimental to the stability of the fork in the tunnel.
2. According to the iterative calculation of the damage to the surrounding rock after the excavation of the fork hole by coupling seepage and stress, without considering the effect of seepage, the damage volume of the surrounding rock is 496.8 m^3^, the total dissipation energy reaches 1.8 T·M, and the maximum damage depth reaches 1.26 m. After considering the effect of seepage, the damage volume of the surrounding rock reaches 567.1 m^3^, the total dissipation energy reaches 1.9 T·M, and the maximum damage depth reaches 1.35 m. This is 14%, 5.6%, and 7.1% higher than that without considering the effect of seepage. As can be seen from the distribution of damage without and with consideration of seepage coupling in Fig. 4a,b, the damage zone around the forked cavern is significantly increased by seepage.
3. When the effect of seepage flow is not considered, the surrounding rocks at the top and bottom of the forkhole are released by the excavation stress, and the displacement deformation is larger, with the amount distributed in 6.2–7.4 mm; the displacement of the surrounding rocks at the waist is relatively small, with the amount distributed in 4.8–5.2 mm (Fig. 5a). After considering the effect of seepage flow, the infiltration heads of the bottom and waist of the forked cavern are larger, and the displacement of the bottom deformation by seepage flow increases to 8.7–9.8 mm and the displacement of the waist increases to 5.4–7.1 mm, while the displacement of the top decreases to 2.9–3.9 mm due to the increased influence of the deformation of the bottom and waist (Fig. 5b), indicating that the coupling effect of seepage flow has an obvious influence on the rocks around the forked cavern.
4. The variation pattern of both the damage zone of the surrounding rock and the displacement at the bottom of the cavern with the number of iterations is: a rapid increase followed by a slow increase, where a number of iterations of 0 means that the coupling effect of seepage and surrounding rock is not considered (Fig. 6).
5. From the stresses in the surrounding rock of the forked cavern, the third and first principal stresses in the top arch and bottom of the forked cavern are about − 1.31 MPa and − 13.56 MPa, and the third and first principal stresses in the waist are about − 0.18 MPa and − 19.25 MPa (Figs. 7a, 8a), and the overall stress deflection in the surrounding rock is about 12.25–19.07 MPa, without considering the effect of seepage. The stress distribution in the surrounding rock is relatively uniform. After considering the coupling effect of seepage flow, the third and first principal stresses at the top and bottom of the fork cavern are approximately − 0.75 MPa and − 16.70 MPa, and the third and first principal stresses at the waist are approximately − 0.19 MPa and − 22.10 MPa, and the overall stress deflection in the surrounding rock increases to 15.95–21.91 MPa (Figs. 7b, 8b). It can be seen that the construction excavation led to the infiltration of external groundwater, which increased the stresses in the surrounding rock around the forked cavern, and the stress deflections increased by approximately 2–3 MPa, indicating that the groundwater infiltration caused the deterioration of the surrounding rock stress state, leading to the increase of the damage zone.

### Calculation of the coupling analysis of water infiltration and stress during lining cracking in operation

**Figure 9:**
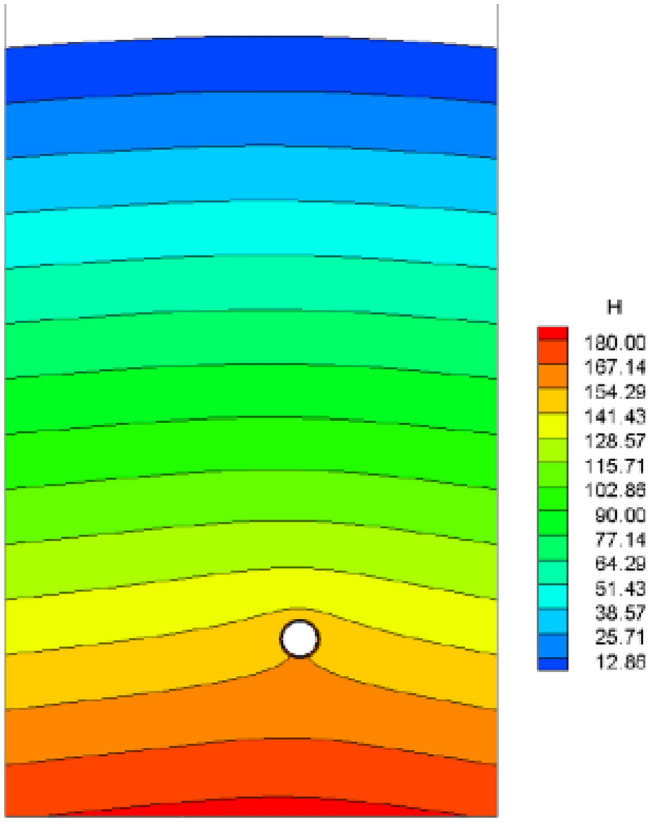
Seepage field at 200 m internal pressure.

**Figure 10:**
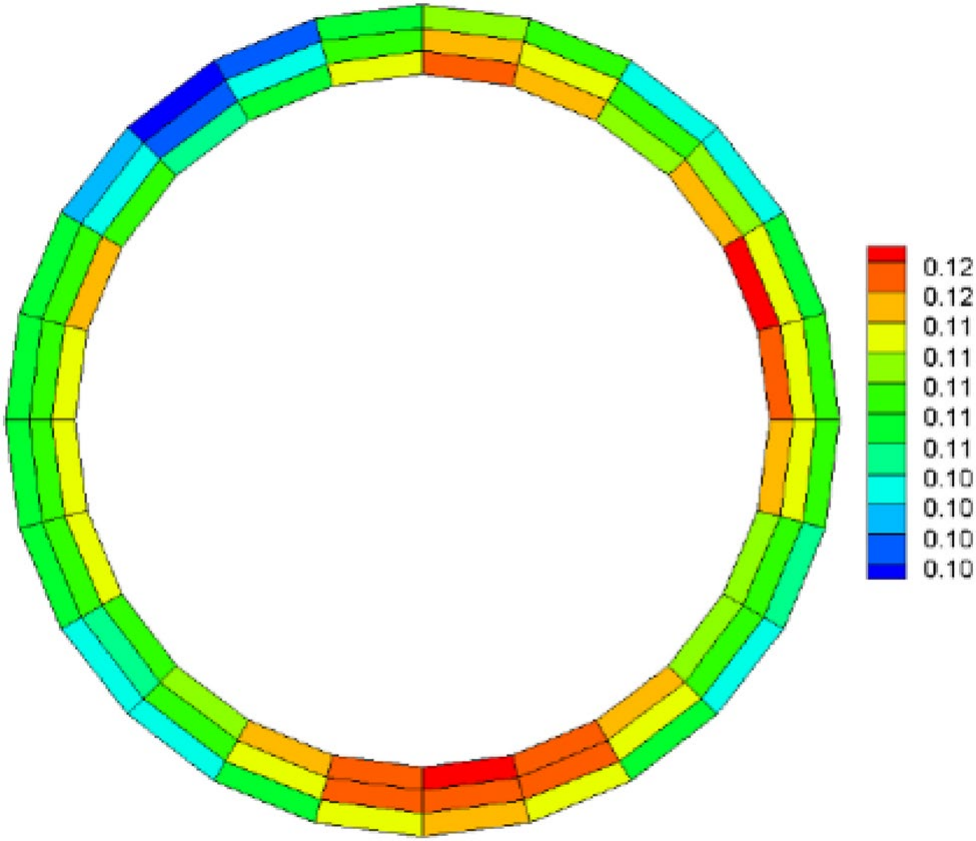
Lining damage factor at 260 m internal pressure.

**Figure 11:**
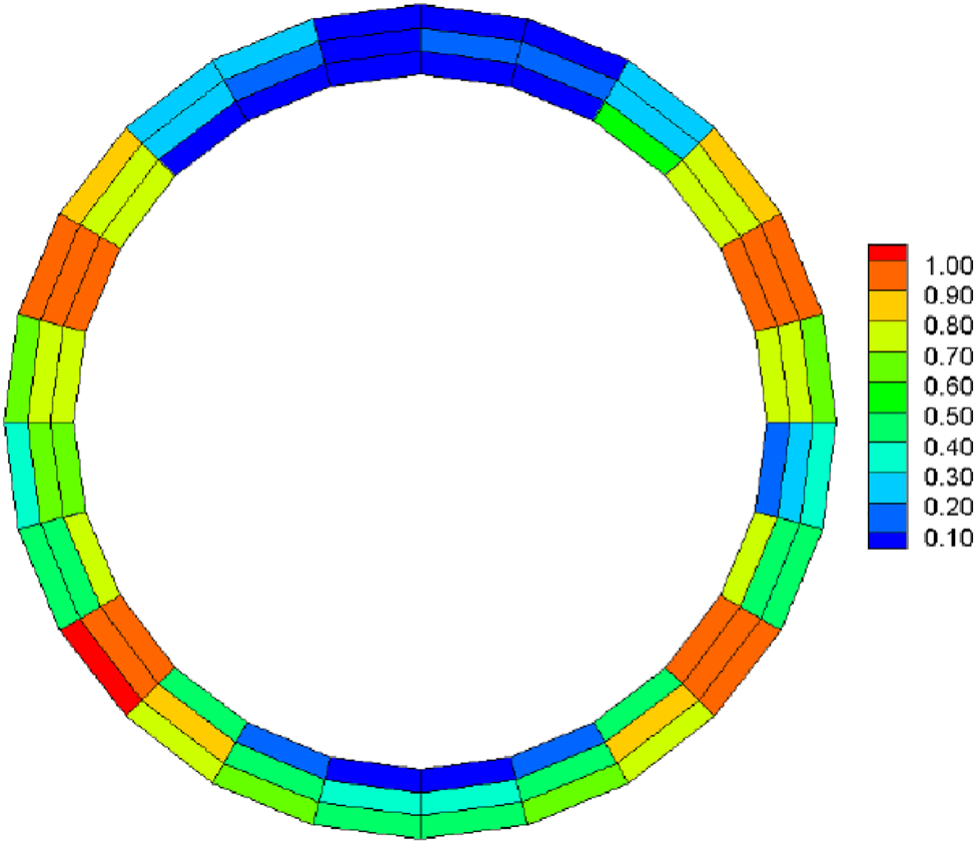
Lining damage factor at 330 m internal pressure.

**Figure 12:**
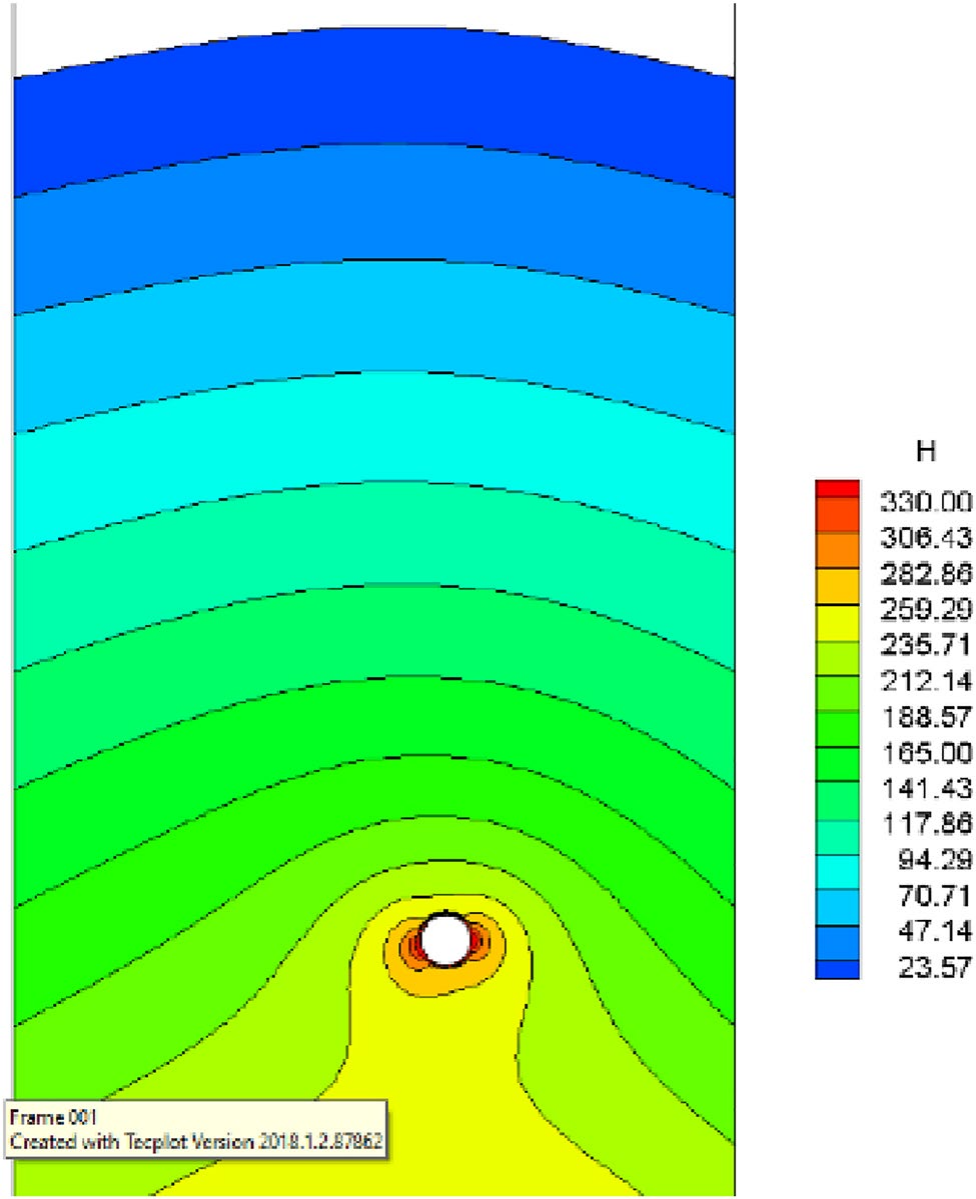
Seepage field at 330 m internal pressure.

**Figure 13:**
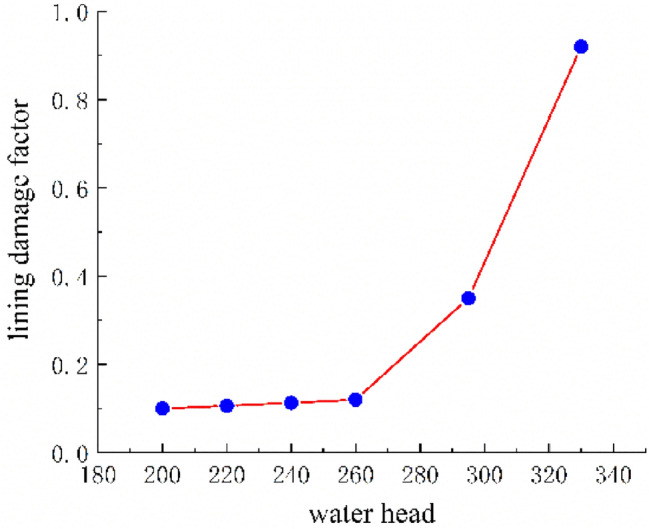
Trend in damage factor.

**Figure 14:**
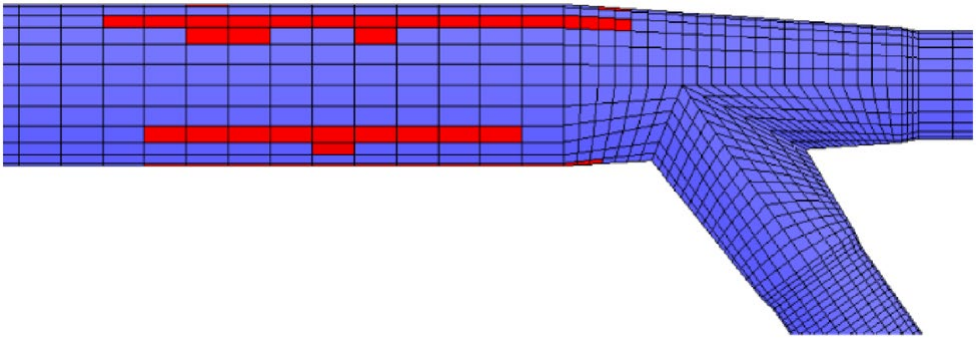
Lining cracking after internal pressure has been applied.

**Figure 15:**
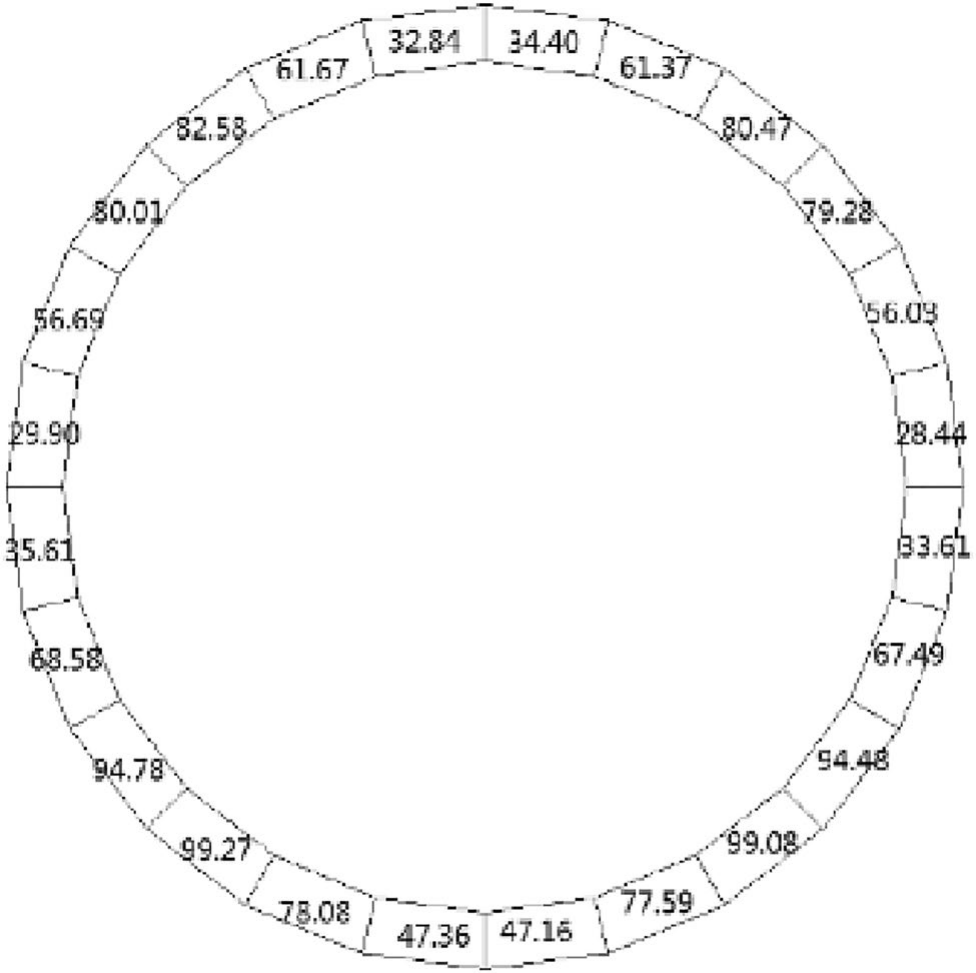
Lining steel stress distribution.

Following the application of the concrete lining of the forked cavern, backfill grouting was performed on the surrounding rock of the forked cavern area in consideration of operational safety, and according to the backfill grouting construction requirements, the outer role of the lining had a pre-pressure effect of 0.39 MPa; the external water head of the forked cavern area was 334.33 m, taking drainage measures into account, and through the coupled iterative analysis of the water pressure inside the ford at 483 m during the operation period was calculated using graded application and coupled iteratively with the seepage flow after the lining was cracked, and the following characteristics can be seen from the iterative calculation process:

1. Due to the grouting pressure and high external water pressure, the pressure applied to the inner wall of the turnpipe lining reaches 200 m; no damage occurs to the turnpipe lining element; at this time, there is no obvious exchange of underground external water in the turnpipe area and internal water in the tunnel; the seepage flow field analysis shows only a little bend in the external water head contour around the turnpipe (Fig. 9); the pressure head borne by the surrounding rock reaches 150 m, indicating that the lining has played a good role in preventing seepage and that the lining is basically balanced by the internal and external water pressure and in a good state of stress.
2. When the water pressure inside the lining reaches 260 m, the waist arch element of the main section at the front of the fork pipe enters a damaged state. With the increase in pressure head, the damage coefficient of the lining element gradually increases, and the damage amount value on the section does not vary much, being basically distributed between *D*_*c*_ = 0.10–0.12 (Fig. 10). The macroscopic crack damage threshold was not reached, and the concrete showed non-linear changes in internal microcrack sprouting and stress–strain.
3. When the pressure at the inner wall of the lining reaches 295 m, the element damage at the waist arch part of the main section at the front of the fork pipe exceeds the threshold value, and with the gradual increase in water pressure, the seepage and stress coupling make the lining crack quickly. The cracks in the inner and outer elements at the two waists of the lining penetrated when the internal water pressure increased to 330 m head due to the influence of the horizontal bifurcation of the forked pipe; the slipping effect between the steel and concrete was obvious, and the damage amount value reached more than 0.90 (Fig. 11). The seepage contours show a clear alternation between internal and external effects; the head contours of the rock around the cave are significantly more dense; and the groundwater level at the centerline of the tunnel is as high as 330 m (Fig. 12). As the internal water pressure increases, the damage factor of the lining unit shows a pattern of slow increase followed by a rapid increase (Fig. 13) This indicates that the coupled calculation of internal and external water infiltration and stress can adequately reflect the high internal water pressure, which can easily lead to the accelerated development of lining cracking due to internal and external water infiltration.
4. When the internal water pressure was applied, several longitudinal extended through cracks appeared in the horizontal pipe section (Fig. 14); the maximum width of the lining cracks was about 0.487 mm, which exceeded the standard of the hydraulic tunnel specification for cracking; the internal steel reinforcement and concrete of the lining showed obvious slippage; the steel reinforcement should not have a large force value under the action of internal and external water pressure, and the distribution was more even; the maximum value was only 99 MPa (Fig. 15). It shows that the lining is infiltrated by internal water, and after the lining is cracked, the surrounding rock bears most of the internal water pressure; and the reinforcement in the lining only bears part of the circumferential force. For the cracked permeable lining, the safety of the lining structure can be guaranteed only if the surrounding rock is reinforced.

## Conclusion

The coupled iterative calculation method of internal water infiltration and lining cracking in the stage of water filling and operation, as well as the coupled iterative calculation method of peripheral rock stress and seepage in the stage of construction and excavation, are proposed in order to address the stress characteristics of high-pressure turnpike pipe under the action of high internal and external water. The pumped storage hydroelectric power plant fork pipe project used the aforementioned technique:

1. The combined effect of the surrounding rock stress and seepage throughout the excavation stage is fully addressed by the linked iterative computation of the surrounding rock and seepage utilizing variable damage stiffness graded loading. The damage zone range, displacement, and stress of the surrounding rock are significantly altered by taking the coupling effect into account as opposed to not doing so.
2. Thin-layer units are used to simulate the radial load transfer between the lining and the surrounding rock, and implicit reinforcement units are used to simulate the circumferential load transfer after the lining cracks, which more accurately represents the load transfer effect of the cracked concrete lining under the influence of high internal water pressure.
3. The high internal water pressure concrete lining can be designed with structural stress reinforcement using the lining water pressure using weighted grading and seepage coupled iterative calculations, which fully responded to the lining force cracking and water seepage coupled to accelerate the destruction of the concrete lining law.

Due to the lack of relevant engineering test results and model tests, the soundness of the proposed theory still needs further verification. Therefore, the design of reasonable model tests to verify the relevant theories is the focus of subsequent research. This paper only considers the influence of fluid–solid coupling on the stability of the surrounding rock under the effect of primary excavation, which is not quite in line with engineering reality. Therefore, the influence of fluid–structure coupling on the stability of the surrounding rock under phased excavation will continue to be studied in the future.

## Data Availability

The datasets used and/or analyzed during the current study available from the corresponding author on reasonable request.
